# Tablet versus paper marking in assessment: feedback matters

**DOI:** 10.1007/s40037-016-0262-8

**Published:** 2016-03-14

**Authors:** Alan Denison, Emily Bate, Jessica Thompson

**Affiliations:** Institute of Education in Medical and Dental Sciences, School of Medicine, Medical Sciences and Nutrition, University of Aberdeen, Aberdeen, UK

**Keywords:** Assessment, Feedback, Technology, OSCE, Undergraduate medical education

## Abstract

**Background:**

The Objective Structured Clinical Examination (OSCE) is a cornerstone in healthcare assessment. As a potential tool for providing learner-centred feedback on a large scale, the use of tablet devices has been proposed for the recording of OSCE marks, moving away from the traditional, paper-based checklist.

**Methods:**

Examiner-recorded comments were collated from successive first year formative and summative OSCE examinations, with paper-based checklists used in 2012 and iPad-based checklists used in 2013. A total of 558 and 498 examiner-candidate interactions took place in the January OSCE examinations, and 1402 and 1344 for the May OSCE examination for 2012 and 2013 respectively. Examiner comments were analyzed for quantity and quality. A tool was developed and validated to assess the quality of the comments left by examiners for use as feedback (Kappa = 0.625).

**Results:**

A direct comparison of paper-based checklists and iPad-recorded examinations showed an increase in the quantity of comments left from 41 to 51 % (+ 10 %). Furthermore, there was an increase in the number of comments left for students deemed ‘borderline’: + 22 %. In terms of the quality of the comments for feedback, there was a significant improvement (*p* < 0.001) between comments left in written-recorded and iPad-recorded examinations.

**Conclusions:**

iPad-marked examinations resulted in a greater quantity and quality of examiner comment for use as feedback, particularly for students performing less well, enabling tutors to direct further learning for these students.

## Essentials

- Using tablet computers instead of paper checklists in clinical exams leads to improved quality and quantity of feedback comments made by examiners.
- Tablet recording of candidate performance in OSCE exams can eliminate ‘missing marks’ that occur with paper recording.
- A novel validated scale for assessing the quality of feedback comments made in an OSCE context is presented.

## Introduction

The Objective Structured Clinical Examination (OSCE) is embedded in the assessment processes of healthcare education [1]. As this assessment tool has extended into postgraduate and early undergraduate contexts, the value of using data acquired during the examination for candidate feedback has also been investigated [2]. In our experience some of the richest feedback comes not from analysis of checklist scores, but from free text comments made by individual examiners.

OSCE marking is most commonly done on machine readable paper sheets, and such comments are typically handwritten. The subsequent process of extracting these comments and presenting them in a timely fashion to candidates can be costly and lengthy, given that a large-scale OSCE may involve several thousand sheets of paper. Technology has been proposed as a tool to support the logistical challenges of large-scale OSCE assessment and for providing a more learner-centred vehicle for providing feedback [3, 4]. This often involves changing from recording performance on a paper-based mark sheet to a computer-based system. Although the purported benefits are attractive, the literature supporting this is modest. In a recent review, Snodgrass [5] identified thirteen articles that discussed electronic methods for practical skills assessment, focussing on health professions. Although the logistical benefits are tangible (such as time saved in processing data and the ability to reuse electronic assessment devices many times), the impact of changing technology on the provision of written comments is uncertain.

This paper reports the impact of the technology change from paper to tablet on the provision of examiner comments. We consider both the quantity and quality of the comments as these comments are often used to provide feedback to our students. It is hoped that our experience will help and empower other healthcare educators considering a similar move.

We aimed to investigate whether the use of an iPad compared with a paper-based marking system affected the number of examiner-student interactions that resulted in a comment being left, the size of the comment (number of words left per comment), and importantly the quality of the comment.

## Methods

We piloted tablet-based recording of candidate performance in formative OSCE assessments in year 1 medicine students in 2011, and extended this in a phased approach to replace paper checklists in formative and summative OSCEs in subsequent years and cohorts of students. Following completion of the assessments, all identifiable student details were removed and the anonymized data separately analyzed as part of an ongoing quality improvement analysis of the transition from paper to tablet recording.

### Ethical considerations

The project was reviewed by the local College Ethics Review Committee. We obtained confirmation that the project and data collection met the criteria for a service evaluation rather than research. This work did not require submission for review on ethics or National Health Service permission.

### App development

In 2011, the University of Aberdeen built an electronic OSCE application (‘app’) for use on iPad devices, containing all of the components of the paper-based marking sheet. Additional functionalities included an inbuilt station timer, a free space section for examiner comments, and a ‘block’ facility to prevent progression to the next candidate if any assessment items had inadvertently not been completed. Following extensive piloting and technical stress tests, the app has now replaced paper-based checklists in the year 1 and 2 OSCEs.

### Assessment periods

We collated the examiner-recorded comments in successive first year OSCE sittings. In 2012, paper-based exam checklists were used for both the first year formative (January) and summative (May) assessments, whilst in 2013, iPad-based exam checklists were used for both the formative and summative OSCE assessments.

All other variables were kept constant, including:

- The formative and summative OSCE station questions, as these were identical for 2012 and 2013.
- Examiner training (with the exception of replacing instructions on completing paper-based checklists and feedback with that of using the OSCE app). Specifically, this included the instruction to write brief free text feedback comments using the on-screen keyboard within an empty box (for iPad OSCE). Previous examiners had been asked to hand write feedback comments within a provided space on the paper checklist.
- The time allocated for the individual OSCE stations and inter-station time.
- The pool of examiners invited to assess the students. Examiners were drawn from a stable and experienced pool that examine throughout the medical curriculum. All had undergone prescribed institutional training in OSCE assessment.

Examiner free-text comments were either written or typed by the examiners for the 2012 (paper-based) and the 2013 (iPad-based) OSCE assessments. These comments were then transcribed and tabulated to facilitate the analysis. The analysis was performed independently by two trained observers, neither of whom had been involved in the OSCEs under evaluation.

### Development and validation of the quality scale

To analyze the quality of the comments for use as feedback, a tool was required to determine the quality of the qualitative comments. In the literature there has been work to assess the quality of written feedback that looks at the feedback written on students’ subject matter knowledge; behaviour; as well as socio-emotive feedback [6]. In 1950, Bales developed a set of categories to help analyze face-to-face interactions [7] while others such as Hyland et al. [8], Brown et al. [9], and Whitelock et al. [6] categorized written feedback but did not explicitly grade it quantitatively according to a scale. However, Glover et al. [10] analyzed science tutor written comments for their a) type and b) depth. The depth analysis was categorized as [10]:

Category 1—An issue is acknowledged, but no corrective advice is offered

Category 2—A correct answer is given and corrective advice is provided

Category 3—The reason why a student’s answer was incorrect is explained, along with feed forward advice

We were unable to identify a validated scale suitable for assessing the quality of comments in an OSCE context. Accordingly, a five-point rating scale was developed (Table 1). We validated the scale for construct validity using the opinions of four independent experts. All were senior faculty staff experienced in providing feedback to students. Inter-rater reliability using two separate blinded independent reviewers, who marked a random sample of 50 comments, yielded a Kappa value of 0.625, demonstrating substantial agreement. Each of the comments left by the examiners was assessed and given a score according to the Feedback Quality Rating Scale. This enabled comparison of the quality of the comments left, for feedback purposes, when using a paper-based OSCE marking system compared with an iPad-based OSCE marking system.

**Table 1:** Feedback quality rating scale

Score	Description	Example
1	Judgmental Non-specific praise Appearance only	*‘Very well done.’* *‘Smartly dressed.’* *‘Good.’*
2	Description of performance **OR** Suggestion for improvement	*‘Very nervous.’* *‘Estimate very low.’* *‘Could slow down slightly on technique.’*
3	Description of performance **AND** Suggestion for improvement	*‘Make sure you have short fingernails, skirt a little on the short side. Good technique for hand washing.’* *‘Use soap! Answer the question. Very nervous. Nice manner despite borderline result.’*
4	Objective appraisal of performance	*‘Poor performance with no demonstration to patient and no attempt to correct poor patient technique nor ensure that the patient can read the meter reading.’* *‘Slightly chaotic, disorganized sequence of examination. However covered most areas and those that were covered were done well. Good rapport with patient.’*
**5**	Objective appraisal of performance **AND** Suggestion for improvement	*‘Done in less than 1.25 min. Lots of white. Did notions but not thoroughly. Suggestion go slower and be more thorough.’* *‘Full marks. One of very few to observe the patient. Telling me what he’s doing but not findings (as many others). Very efficient, could be a bit gentler e.g. turning arms over. Told patient to breathe consistently—very good!’*

## Results

A total of 558 and 498 examiner-candidate interactions took place in the January OSCE examinations, and 1402 and 1344 for the May OSCE examination for 2012 and 2013, respectively. This included a total number of 82 examiners across all examinations.

### Quantity of feedback

Between the formative examinations (January 2012 and January 2013) there was a 2 % decrease in the number examiner-students interactions that resulted in a comment being left by the examiner (Table 2). For the summative (May 2012 and May 2013) assessments, there was a 15 % increase. Overall, a comparison of the paper-based checklist examinations (January and May 2012) with the iPad-recorded assessments (January 2013 and May 2013) showed an increase in the number of comments left from 41 to 51 % (+ 10 %), but a decrease in the mean number of words left in each comment, from 16 to 13 words when using paper-based checklists compared with iPad-recorded checklists (Table 2).

**Table 2:** Quantities of comments provided in formative and summative assessments, 2012 and 2013

Exam style	Date	Number and percentage of examiner-student interactions where a comment was made (%)	Mean number of words	Standard deviation	Total number and percentage of examiner-student interactions where a comment was made (%)	Total mean number of words
Paper- based	Jan 2012	558 (68)	19	14	1960 (41)	16
	May 2013	1402 (30)	13	10		
iPad-based	Jan 2012	498 (66)	15	11	1842 (51)	13
	May 2013	1344 (45)	12	8		

### Quantity related to global score

The quantity of feedback left in each examination was further analyzed, to determine whether there was any correlation between the number of comments left and the global score obtained by the candidate. This is shown in Table 3.

**Table 3:** Quantity of feedback received related to global score

Total number of students receiving Global Score		Written (Jan and May 2012)	iPad (Jan and May 2013)
5 ‘Excellent’	Number and percentage of students receiving feedback	111 (*n* = 299) 37 %	148 (*n* = 270) 55 %
	Mean number of words of feedback	12	9
4 ‘Highly satisfactory’	Number and percentage of students receiving feedback	212 (*n* = 622) 34 %	235 (*n* = 539) 44 %
	Mean number of words of feedback	14	13
3 ‘Satisfactory’	Number and percentage of students receiving feedback	296 (*n* = 736) 40 %	317 (*n* = 724) 44 %
	Mean number of words of feedback	17	13
2 ‘Borderline’	Number and percentage of students receiving feedback	113 (*n* = 219) 52 %	179 (*n* = 242) 74 %
	Mean number of words of feedback	18	15
1 ‘Unsatisfactory’	Number and percentage of students receiving feedback	74 (*n* = 84) 88 %	58 (*n* = 67) 87 %
	Mean number of words of feedback	18	19

As mentioned earlier, the number of words per item of feedback decreased when examiners used iPad-based marking compared with paper-based marking, but the percentage of students receiving a feedback comment increased when using an iPad. This increase was by 18 % for students receiving a global score of 5 (Excellent) and by 22 % for students receiving a global score of 2 (Borderline). Importantly, the percentage of students receiving feedback when they obtained a global score of 1 (Unsatisfactory) did not decrease (88 % for paper-based and 87 % for iPad-based).

### Quality of the comments as feedback

To assess the quality of the comments left as feedback, the five-point Feedback Quality Rating Scale was used (Table 1). The mean and standard deviations for the Feedback Quality Rating Scale score obtained for each of the four examination sessions are shown in Table 4. The mean Feedback Quality Score using the iPad was 2.41, compared with 2.18, using paper-based marking systems.

**Table 4:** Mean and standard deviation of the iPad versus written OSCE comments, when rated by the Feedback Quality Scale

	Comment Feedback Quality Score					
	Jan 2012 (written)	Jan 2013 (iPad)	May 2012 (written)	May 2013 (iPad)	Overall Written	Overall iPad
Mean	2.36	2.56	2.02	2.32	2.18	2.41
SD	0.95	1.02	0.56	0.81	0.78	0.89
N	381	330	425	607	806	937

To determine whether this difference was statistically significant, the Students t-test was used, which demonstrated that this increase in the Feedback Quality Score, when using an iPad-based marking system, was statistically significant for both the formative (*p* = 0.0064) and summative (*p* < 0.0001) examinations. Similarly, when written and iPad exams were directly compared using the Students t-test, there was a significant difference (*p* < 0.0001).

Another important observation was that there was a reduction in the number of occasions when examiners left only non-specific praise for candidates (Feedback score 1) when using the iPad-based marking system. In fact, the number of instances was exactly halved in both examinations (the formative and summative) when using the iPad compared with the paper-based exams (from 68 to 34 instances of non-specific praise).

### Missing marks

There were no missing marks for the iPad-based OSCE examinations, compared with a total of 115 missing marks in the 2012 exam.

## Discussion

The transition from paper-based to computerized recording of candidate performance brings challenges and risks, but also anticipated and unanticipated benefits. In this work, we found that the adoption of new technology led to the provision of improved quantity and also quality of comments left by OSCE examiners, which are used for the provision of student feedback. The electronic nature of the comments may shorten the time interval before feedback can be provided to students, and may also facilitate the future provision of feedback to all (rather than just some) candidates.

There was a reduction in the number of occasions where examiners left only non-specific praise for candidates using the iPad devices. While positive comments allow candidates to know something was done correctly, there are risks in using this type of language. Ambiguous comments, such as ‘good effort,’ do not inform the recipients which aspects of the performance were of value [11]. In a study of helpful and unhelpful feedback techniques, Hewson and Little [12] describe an incident deemed unhelpful due to the non-specific praise delivered, establishing the importance of ensuring feedback is based on observations, including when praising someone. There was a difference in feedback provision between the formative and summative contexts when using iPads. The explanation for this is uncertain. Although a learning curve associated with the iPad could influence this, a more plausible explanation is that the nature of formative and summative assessments differs, and that examiners may purposefully provide more detailed feedback at formative encounters, and restrict their comments to weaker candidates at summative encounters.

The reduction in the overall number of comments left may be complex and influenced by several factors including examiner training, overall competence of the candidates and the time constraints of individual stations.

A key strength of the work is that the same test items were used for both paper- and tablet-marked OSCEs in separate academic years and cohorts. This allowed us to interrogate candidate/examiner data for nearly 4000 OSCE stations, and be confident that the introduction of the new technology did not weaken the provision of quality or quantity of examiner comments.

Although the OSCE station material was identical between assessments, the individual examiners were not, and the initial examiners using tablet devices might have been self-selecting. However, we purposefully used our standard recruitment measures for examiners for the iPad OSCEs, since we wished to ensure that the technology would work irrespective of the seniority and background of examiners. It is also possible that with the new technology, the stability and rise in the comments provided reflected not the design of the app, but bias due to early adoption of new technology [13], with examiners interacting more with the iPad than they might otherwise do with paper. However, tablet computers have an increasing footprint in healthcare service contexts, and as such the novelty factor is likely to be modest. We also acknowledge that other factors such as cohort effect, administration differences and slight curricular changes would inevitably make identical comparisons between the two cohorts impossible.

A further potential limitation of this study is that for both iPad-based OSCE mark schemes, the comments that have been analyzed are from a section entitled ‘Comments’. Whilst these are used for feedback purposes during student support interviews, these comments are not necessarily written by the examiners with the intent that they will be directly given to the students. Nevertheless, we have demonstrated the improvement in the quality of the comments for use as feedback, using an iPad compared with a paper-based system. Another next step could be to change the title on the iPad marking system to ‘Candidate feedback’ instead of ‘Comments’ and encourage the examiners at the pre-examination briefing to leave ‘Feedback for the candidates’ rather than emphasizing that ‘their comments will be helpful’ when reviewing the students’ OSCE mark sheets. Some examiners may assume that this is what the ‘Comments’ are for, but making this explicit may further enhance the feedback quantity and quality received.

Although several qualitative frameworks exist to analyze written feedback, we judged that none were suitable for the context of brief comments in an OSCE. The rating scale used to assess the quality of the free-text comments was developed and piloted in an iterative manner with independent testing of reliability to maximize its utility. The scale took into consideration several aspects of feedback that were desirable or conversely could be considered unhelpful. Further validation would be valuable.

A further strength of using tablet devices was that missing data (i.e. unmarked items on the checklist) never occurred, due to the design of the app. Although the number of marks missed during the paper-marked assessments was small, this probably underestimates the true incidence since examiners may be prompted (or remember) to complete unmarked items in advance of later administrative checking systems. Ultimately, less missing marks results in a higher quality of data.

An under-anticipated benefit of using tablet devices was the very considerable saving both in administrative staff time and cost of single-use specialist computer-readable paper sheets. While this has been described previously [3], the advantages were well in excess of our expectations. Raw results can be available for analysis within minutes following the final OSCE bell. Tablet devices are individually expensive, but are reusable and are deployed in multiple different assessment and other contexts in our institution.

The exams under scrutiny in this work were first year OSCEs and the stations were straightforward. Careful analysis of our planned further deployment of tablet marking in more advanced stations in senior years will be required, to determine if tablets will remain a suitable vehicle for capturing comments for use as feedback, which may be more complex than in early years curricula.

In conclusion, the use of tablet devices in OSCE assessments is associated with improved examiner comment for use as feedback compared with the traditional, paper-based data capture using computer read mark sheets.

### Source(s) of support in the form of grants

University of Aberdeen Medical Education Summer Teaching Bursary.
